# Retraction: Role of SDF-1α/CXCR4 signaling pathway in clinicopathological features and prognosis of patients with nasopharyngeal carcinoma

**DOI:** 10.1042/BSR-2017-0144_RET

**Published:** 2024-04-24

**Keywords:** Nasopharyngeal carcinoma, Signaling pathway, Stromal cell derived factor receptor-4, Stromal cell derived factor-1α

This article is being retracted from Bioscience Reports at the request of the Editor-in-Chief and the Editorial Board following receipt of a notification from a reader, alerting the Editorial Board to multiple images within the article that seem to appear in other papers by different authors. Specifically:

- Figure 1A part of which appears as Figure 1 IGF1R Nasopharyngeal carcinoma panel from Wang et al 2018 (DOI: 10.1002/jcb.26354) [retracted], Figure 1A Nasopharyngeal carcinoma panel from Cheng et al 2018 (DOI: 10.1186/s12935-018-0605-0), and Figure 2A NPC tissue panel from Cheng et al 2018 (DOI: 10.3233/CBM-181459) [retracted]
- Figure 1B part of which appears as Figure 1 TGFβRI Adjacent normal tissue panel from Ma et al 2017 (DOI: 10.1159/000481871) [retracted]
- Figure 2B part of which appears as Figure 2A VEGF NPC tissues panel from Chen et al 2020 (DOI: 10.1002/jcp.29382)

The authors have been contacted with regards to the retraction and have not responded to the Journal's queries or the concerns raised. Given the extent of the issues raised, the Editorial Board stand by the decision to retract the article.

